# Immunogenicity in Fabry Disease: Current Issues, Coping Strategies, and Future Directions

**DOI:** 10.3390/biomedicines14020343

**Published:** 2026-02-02

**Authors:** Andrea Matucci, Sandro Feriozzi, Elena Biagini, Mario Mangeri, Matteo Accinno, Michael Diomaiuti, Raffaello Ditaranto, Cristina Chimenti, Calogero Cirami, Francesca Graziani, Antonio Pisani, Alessandra Vultaggio

**Affiliations:** 1 Immunoallergology Unit, Careggi University Hospital, 50134 Florence, Italy; 2 Nephrology Unit, Fondazione Policlinico Universitario Campus Bio-Medico, 00128 Rome, Italy; 3 Cardiology Unit, Department of Experimental, Diagnostic and Specialty Medicine, Alma Mater Studiorum, University of Bologna, 40126 Bologna, Italy; 4 Nephrology and Dialysis Department, Belcolle Hospital, 01100 Viterbo, Italy; 5 Department of Experimental Medicine, University of Florence, 50121 Florence, Italy; 6 Department of Clinical, Internal, Anesthesiology and Cardiovascular Sciences, Sapienza University, 00185 Rome, Italy; 7 Nephrology, Dialysis and Transplantation Unit, Careggi University Hospital, 50134 Florence, Italy; 8 Non-Invasive Cardiovascular Diagnostic Unit, Fondazione Policlinico Universitario A. Gemelli IRCCS, 00168 Rome, Italy; 9 Department of Public Health, Chair of Nephrology, Federico II University of Naples, 80131 Naples, Italy

**Keywords:** anti-agalsidase antibodies, ADAs, infusion-related reactions, enzyme replacement therapy, ERT, Fabry disease, immunogenicity

## Abstract

Fabry disease (FD) is an X-linked systemic lysosomal storage disease caused by mutations in the galactosidase-α (*GLA*) gene, which encodes the α-galactosidase A (α-AGAL) enzyme. FD can lead to serious complications, including early death, if left untreated. For over 20 years, enzyme replacement therapy (ERT) based on the use of agalsidase-α and agalsidase-β has been the standard treatment for FD, alongside new molecules that have enriched the therapeutic armamentarium and others that are being tested to expand it further. Unfortunately, ERT can be associated with the formation of inhibiting antidrug antibodies (ADAs), which impact ERT clinical efficacy and have consequences affecting safety and therapeutic adherence. A group of FD specialists discussed the problem of immunogenicity in FD, analyzing the most recent literature and the strategies that are currently being used to address it. Once formed, fluctuating levels of ADAs persist and have an impact on the clinical picture and prognosis of the disease that is still the subject of lively scientific debate. The critical nature of ADAs is demonstrated by their ability to bind to the enzyme, increasing drug clearance while forming immune complexes that can build up in the tissues causing chronic inflammation that aggravates the progression of the disease and affects the onset of acute reactions after the infusion, impacting therapeutic adherence. Although similar in their therapeutic mechanism, agalsidase-α and agalsidase-β differ in their production process, with resulting differences from a pharmacokinetic and pharmacodynamic point of view and diverse immunological implications: despite showing rather overlapping efficacy outcomes, agalsidase-α demonstrates a better tolerability profile, with a lower frequency of ADAs, than agalsidase-β. Given the extreme variability of the clinical picture, it is crucial for optimal FD management that the most appropriate molecule is chosen by taking into account the unique immunological risk profile of each single patient, and particular attention should be paid to naïve subjects by periodic measurement of ADAs during therapy and cross-referencing data to correlate serological and clinical patterns.

## 1. Introduction

**Figure 1:**
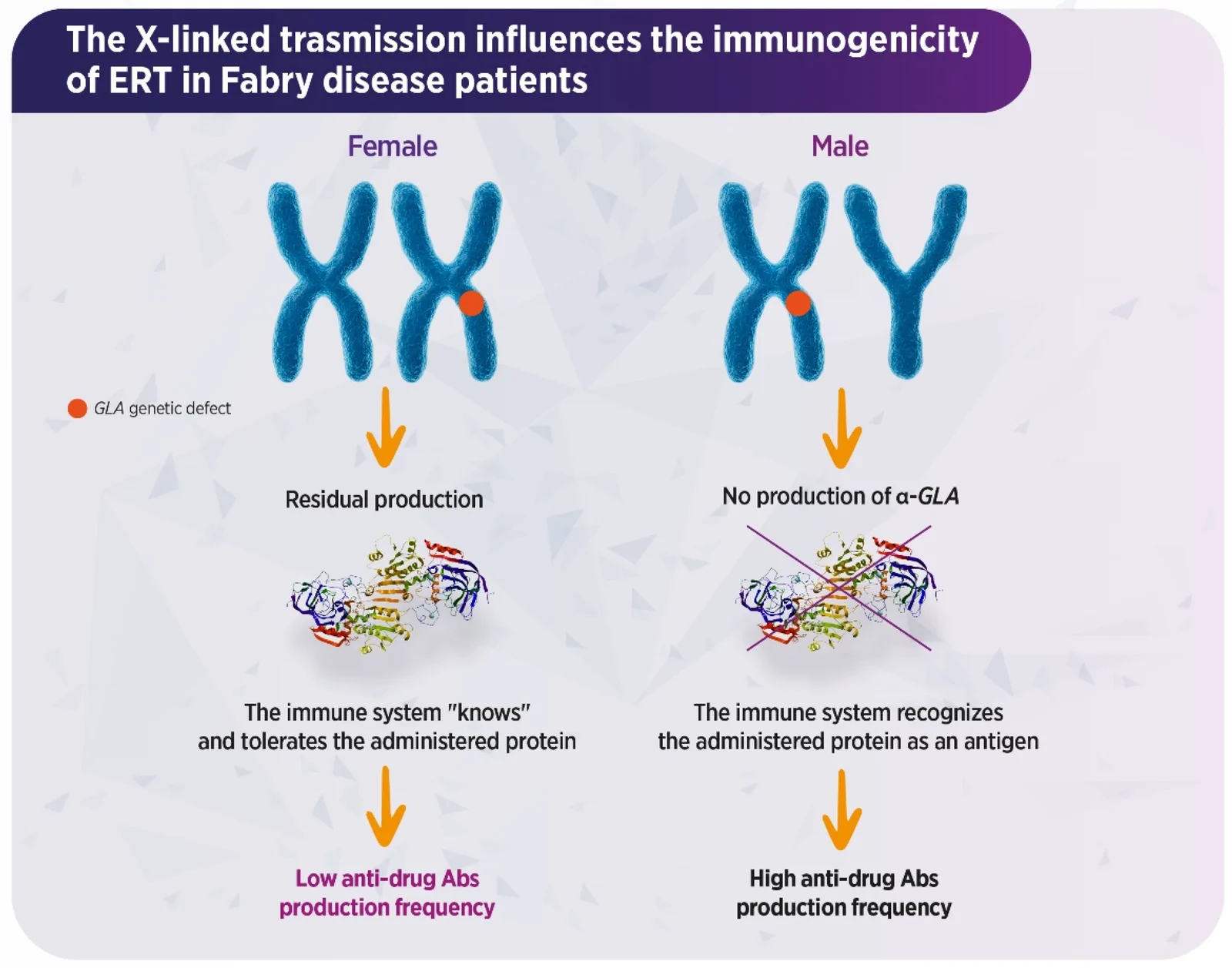
How X-linked transmission influences immunogenicity in Fabry disease. galactosidase-α (*GLA*) gene, mutation on the X chromosome results in loss of enzyme production and the absence of the protein in the serum of males; therefore, the immune system will not tolerate a foreign antigen. Ab, antibody; α-GAL, α-galactosidase A. In females, X-chromosome skewed inactivation is associated with variable serum enzyme levels, which determine tolerance to the infused enzyme.

Fabry disease (FD; OMIM 301500) is a rare, X-linked lysosomal storage disease (LSD) caused by mutations in the galactosidase-α (*GLA*) gene leading to absent or reduced activity of the α-galactosidase A (α-AGAL) enzyme. This results in systemic intracellular accumulation of neutral glycosphingolipids (GSL), most notably globotriaosylceramide (Gb3) and its deacylated form globotriaosylsphingosine (lysoGb3) [1,2]. Gb3 accumulation leads to organelle disruption, with consequent apoptosis, inflammation, and increased oxidative stress (OS), which cause progressive multi-systemic damage that is clinically evident at the level of the heart, kidneys, and brain [3]. Cardiac involvement such as hypertrophic cardiomyopathy and cardiac arrhythmias represent some main prognostic determinants. Additionally, progressive renal failure and recurrent cerebrovascular events significantly limit quality of life (QoL) and life expectancy in affected patients [4]. Male patients with the classic phenotype have little to no *GLA* activity and typically develop early-onset symptoms, while non-classic males who have residual *GLA* activity present milder and late-onset manifestations [5]. On the other hand, female patients who are heterozygous for *GLA* mutations can display a broad spectrum of symptoms or remain entirely asymptomatic [5] (Figure 1).

Current FD treatment is based mostly on ERT using agalsidase-α produced in human fibroblasts and agalsidase-β prepared by means of Chinese hamster ovary (CHO) cells [6,7]. More recently, pegunigalsidase-α, a polyethylene glycol (PEG)ylated molecule produced using tobacco plant cells with an increased plasma half-life, has been approved, while chaperone therapy with migalastat represents an additional therapeutic option, which is limited to patients with amenable mutations [8,9]. A growing body of evidence shows that male patients with classical FD develop antidrug antibodies (ADAs) following ERT, even though ADA formation has also been described in females and in non-classical patients with milder forms of the disease [10]. ADAs formation has been associated with two significant clinical events, i.e., infusion-related reactions (IRRs) and reduction of ERT effectiveness, which affect treatment adherence and efficacy.

The aim of this review is to discuss the different factors which influence ERT-related immunogenicity and its impact on FD clinical outcomes, through a structured and rational approach based on questions whose answers were derived by critical assessment of the current scenario in the light of the most recent literature on the subject.

## 2. Challenging Questions and Evidence-Based Answers

### 2.1. Which Factors Are More Likely to Predispose to ADAs Development During ERT Administration in FD Patients?

**Figure 2:**
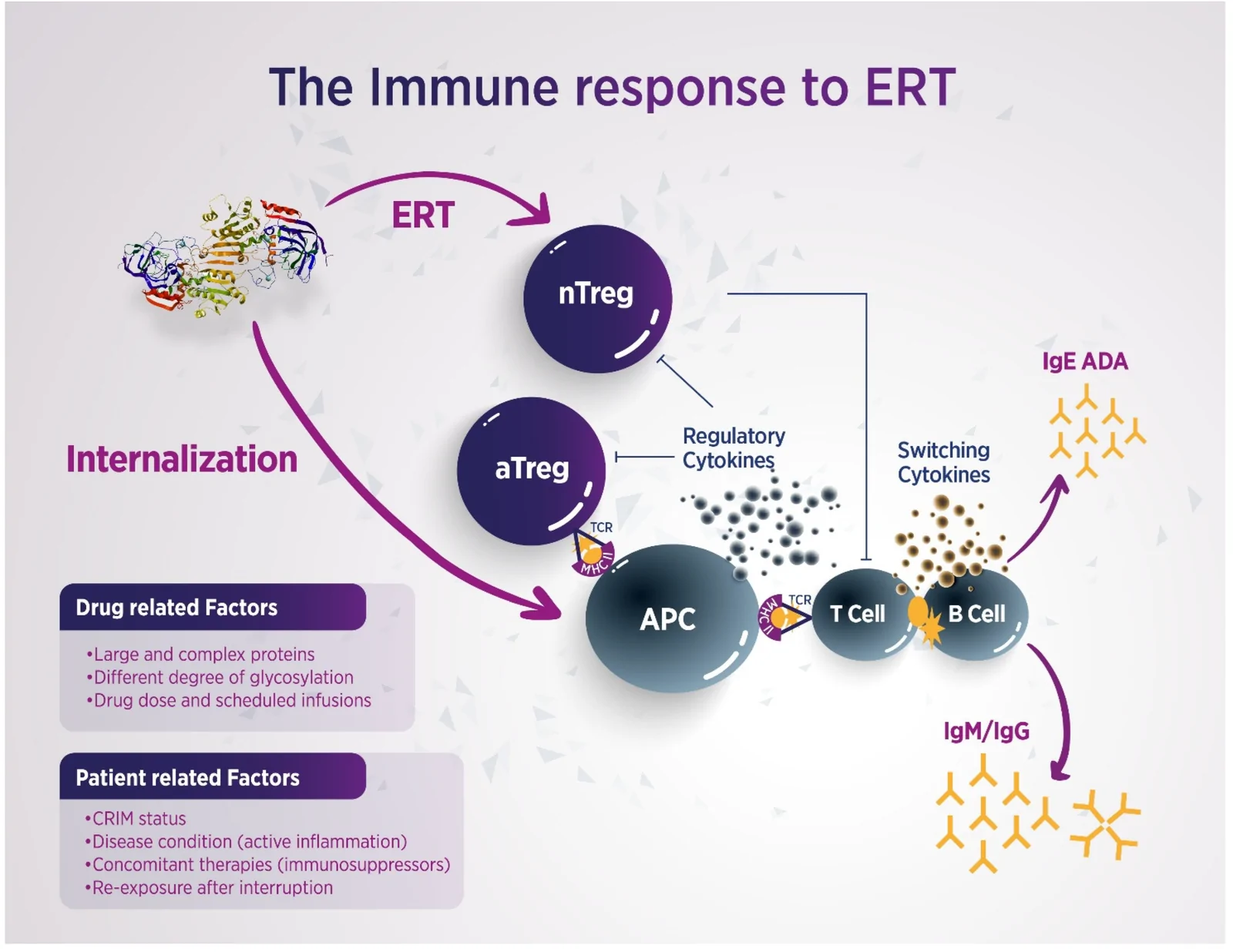
The immune response to ERT in FD. The enzyme is captured by APCs, which display enzyme-derived peptides on their membranes in the context of Class II molecules of the MHC. The displayed peptides are recognized by specific T lymphocytes, and B lymphocytes produce antibodies of the IgM class, followed by IgG or IgE. The immune response can be modulated by naturally occurring nTregs or aTregs via membrane contact or by regulatory cytokines. ERT, enzyme replacement therapy; CRIM, cross-reactive immunologic material; APCs, antigen-presenting cells; MHC, major histocompatibility complex; nTregs, natural regulatory T cells; aTregs, adaptative regulatory T cells; immunoglobulins (Ig) TCR, T cell receptor; ADAs, anti-drug antibodies.

The development of ADAs following the use of bio-therapeutics to treat different conditions is associated with a number of drug- and patient-related factors. In FD patients undergoing ERT, immunogenicity might be triggered by the enzyme dose, the frequency of administration, the cell line in which the molecule is produced (i.e., human fibroblasts versus CHO cells), the age of the recipient, and the mutation type [11]. The recombinant proteins infused in ERT are recognized as foreign antigens by the recipient’s immune system, processed by antigen-presenting cells (APCs), and then displayed to specific T helper cells that collaborate with B lymphocytes, leading to the development of an expected humoral response, particularly in patients with cross-reactive immunologic material (CRIM)-negative status, hemizygous male patients and a few homozygous female patients with unfavorable skewed inactivation [12,13,14] (Figure 2). α-AGAL is absent in hemizygous males and may be present in females with skewed inactivation, leading to a more frequent and severe immune reaction, whereas in CRIM-positive subjects, the antigenic sites of the infused enzyme are recognized as self, and the immune reaction does not occur [14]. Indeed, in classical patients with missense mutations, small amounts of the protein are still produced, usually resulting in central tolerance induction, while in subjects with large deletions or early frameshift mutations, the protein will either not be produced at all, or it will be truncated, likely causing an immunological response towards the exogenously administered enzyme [15]. ADAs production is described in 40–70% of male patients undergoing ERT, as these cohorts have little to no native enzyme due to the underlying genetic defect, and their immune system naturally reacts to an alien antigen; conversely, most female patients develop fewer antibodies due to their heterozygous status and residual enzyme activity [16]. Immunogenicity findings observed in FD during the course of ERT are aligned with an association between mutation type and ADA development in Pompe disease and hemophilia [17,18]. Indeed, van der Venn et al. reported that having a nonsense or frameshift *GLA* mutation and undergoing treatment with agalsidase-β were associated with an increased risk of ADA development in a large cohort of classical male patients [10] (Table 1).

### 2.2. Is There Any Difference in ADA Formation Related to the Type of ERT Used?

The immunogenic potential of ERTs in FD is influenced by the manufacturing process, particularly the cell line used for enzyme production. While the primary amino acid sequence of all approved ERTs mirrors that of the native human α-GAL, differences in post-translational glycosylation patterns contribute significantly to their immunogenic profiles [22]. Agalsidase-α, produced in human fibroblasts, exhibits a glycosylation pattern that is more similar to the human endogenous enzyme, and it is associated with lower immunogenicity compared to agalsidase-β, which is derived from CHO cells. The latter presents increased phosphorylation and fully sialylated oligosaccharides, which are structurally divergent from human proteins, potentially enhancing its immunogenic profile [23,24]. Notably, higher sialylation improves the targeting of agalsidase-β to mannose-6-phosphate (M6P) receptors in affected organs, protecting the enzyme from hepatic clearance and increasing its bioavailability [25,26]. However, agalsidase-β contains N-glycolylneuraminic acid (NGNA), a non-human sialic acid which is absent in human tissues, and it is associated with heightened immunogenicity [27]. Although increased M6P expression induced by agalsidase-β facilitates uptake by target tissues such as the kidney and heart, it may also promote its distribution to non-target sites, such as the liver. Animal studies have shown that dose-dependent increases in α-GAL activity occur in various organs following intravenous administration of both agalsidase-α and agalsidase-β [28]. Moreover, there is a structural difference in the carboxyl (C)-terminal sequence of the two ERT, since agalsidase-α lacks a terminal leucine residue which is found in agalsidase-β, and this deletion may influence enzyme activity, although current data do not allow definitive conclusions to be drawn regarding either its functional impact or any correlation with immunogenicity [29,30]. In the absence of large-scale comparative studies on the immunogenicity of both ERTs in treatment-naïve patients and international standards for assessing ADAs titers, the most compelling comparative data on immunogenicity come from two randomized controlled trials (RCTs) by Vedder et al., which demonstrated a significantly higher incidence of ADAs in patients treated with agalsidase-β than those receiving agalsidase-α [31,32]. These findings were corroborated by Arends et al., who reported an increased risk of neutralizing ADA formation in males treated with agalsidase-β [13].

Post-marketing surveillance (PMS) data further support these findings, indicating that approximately 70% of agalsidase-β-treated patients develop IgG antibodies, compared to only 24% of those treated with agalsidase-α, and these substantial differences might be due to the different origin of the two molecules, their dosage, or both [19,33]. In a recent long-term surveillance study, Arakawa et al. observed that only 10.3% of classic male patients (6 out of 58) developed ADAs against agalsidase-α, which is a significantly lower incidence than that reported in earlier studies [20].

Contrary to initial expectations, pegunigalsidase-α also demonstrates some degree of immunogenic potential, since its non-human glycosylation profile and the PEG moiety can both induce antibody responses, with ADAs reported in about 30% of treated individuals [34]. Additionally, pre-existing or treatment-induced anti-PEG antibodies have been implicated in increased drug clearance and reduced therapeutic efficacy across multiple PEGylated biologics [35]. In a recent analysis by Lenders et al., among 13 patients (i.e., 11 previously treated, 2 naïve), pre-existing neutralizing ADAs were found to negatively influence the pharmacokinetics of pegunigalsidase-α [34]. These patients showed reduced plasma half-life and peak enzyme activity, along with the formation of immune complexes in four individuals who were already positive for anti-AGAL antibodies, three of whom showed increased titers during follow-up, while no de novo formation of anti-PEG or anti-AGAL antibodies was observed in this cohort [34]. Nevertheless, it is vital to analyze larger case series with longer follow-ups in order to state whether anti-PEG ADAs have any clinical impact, since FD trajectories are unpredictable and varied and can take a long time to manifest. Importantly, IgG antibodies generated against agalsidase-α or agalsidase-β can cross-react with both formulations, mutually inhibiting in vitro enzymatic activity from 65% up to 95% [25]. The issue of cross-reactivity is especially relevant with the advent of pegunigalsidase-α, even though PEGylation appears to partially shield immunogenic epitopes, resulting in lower affinity and reduced inhibitory capacity of pre-existing ADAs compared to native forms [36] (Table 2).

### 2.3. What Is the Relationship Between ADA Production and IRRs?

The occurrence of IRRs in FD patients represents a clinical concern linked to ERT-related immunogenicity. IRRs are more frequent in ERT-naïve patients at the beginning of treatment, especially in male subjects, and their clinical presentation spans from mild to life-threatening manifestations [21]. According to the FOS (Fabry Outcome Survey), mild IRRs occur in approximately 13% of patients infused with agalsidase-α, while RCTs report mild to moderate IRRs in up to 60% of subjects receiving agalsidase-β and in about 20% of those treated with pegunigalsidase-α [37,38,39,40]. IgE antibodies, which are usually associated with type I hypersensitivity reactions, are not often found in FD patients who develop IRRs, suggesting that IgE-dependent immune pathways are not the only responsible mechanisms in these cohorts [23]. On the other hand, murine models showed that anaphylaxis also occurs through an IgG-dependent mechanism in which FcγRIII (CD16), macrophages, and basophils are involved, with platelet activating factor (PAF) as a major mediator [41]. Moreover, the occurrence of severe IRRs in patients treated with biological agents such as anti-TNF-α infliximab who are negative for IgE and IgM ADAs strongly suggests a role for IgG ADAs in the induction of anaphylaxis [42]. This observation is in agreement with data observed in FD patients who develop anti-αAGAL IgG, who are more likely to experience IRRs than seronegative cohorts [43].

### 2.4. What Is the Impact of Anti-Agalsidase Antibodies on FD Biomarkers and Overall ERT Effectiveness?

Although the potent in vitro inhibition of enzymatic activity by both agalsidase-α and agalsidase-β in the presence of IgG-positive sera assessed by ELISA assay is well-established and ranges between 65% and 95% due to cross-reactivity, the effects of ADAs on intracellular enzyme function remain an expanding area of investigation [25]. Neutralizing ADAs directed against exogenous AGAL predominantly belong to the IgG1 and IgG4 subclasses. These antibodies can bind epitopes located at uptake-relevant domains, i.e., amino acid positions N139, N192, and N215, making them inaccessible to M6P receptors, especially during infusion [16,44]. Anti-agalsidase-β antibodies inhibit enzyme activity in cultured fibroblasts derived from FD patients, and high anti-agalsidase-β IgG titers are significantly associated with the presence of Gb3 deposits in the endothelial cells of dermal capillaries [45,46]. A similar detrimental impact has been observed with agalsidase-α in patients exhibiting high and persistent ADA titers, as shown by a smaller decrease in plasma lyso-Gb3 levels [47]. The interpretation of Gb3 levels in plasma and urine remains challenging, due to the fact that the lysosomal delivery of agalsidase-β to podocytes is not solely dependent on M6P receptor binding, but also involves additional membrane glycoproteins such as megalin and sortilin, and the presence of specific neutralizing antibodies may impair these uptake pathways [48]. In a cohort of classical FD patients receiving either agalsidase-α or agalsidase-β, following an initial decrease in both ADA-positive and ADA-negative subjects, plasma lyso-Gb3 levels remained significantly elevated over a 6-year follow-up period only in ADA-positive individuals, whereas urinary Gb3 decreased only in ADA-negative patients [16]. The uptake of infused AGAL via M6P receptors leads to enhanced lysosomal AGAL activity and subsequent substrate clearance. However, ADAs can reduce the therapeutic efficacy of ERT by altering its pharmacodynamics. For instance, Fcγ receptor-expressing cells, such as macrophages, may recognize ADA-drug immune complexes, accelerating enzyme clearance from circulation [25]. Given the polyclonal nature of ADAs in FD, which recognize multiple epitopes, a multifaceted mechanism involving both enzyme inhibition and immune-mediated clearance likely contributes to reduced therapeutic benefit and potential safety concerns [49]. Although not yet confirmed in FD, studies from Pompe disease suggest that large immune complexes formed by ADAs and therapeutic proteins could activate the complement cascade, leading to membranous nephritis [50]. ADAs that do not interfere directly with AGAL activity or uptake are classified as “non-neutralizing,” but they may still influence the pharmacokinetics, intracellular trafficking, and conformational integrity of the administered enzyme. Overall, ADA-positive FD patients, regardless of whether the antibodies exhibit inhibitory or clearance-promoting properties, have been associated with reduced AGAL efficacy, impaired endothelial enzyme uptake, and diminished intracellular activity, which cumulatively result in suboptimal clinical response and worse long-term outcomes [51]. While some studies have reported no significant correlation between ADAs and clinical endpoints such as estimated glomerular filtration rate (eGFR) decline or changes in cardiac mass, others clearly demonstrate that ADA-positive individuals exhibit more severe disease [51,52]. In vitro experiments further corroborate the negative impact of ADAs inhibiting AGAL activity, showing that they can neutralize AGAL enzymatic activity, hinder cellular uptake, and alter the pharmacokinetic profile of recombinant enzymes in a titer-dependent manner [53]. The formation of ADAs has also been linked to incomplete substrate clearance in endothelial tissues and, in some cases, substrate re-accumulation following initial reduction, potentially exacerbating disease severity [54]. Male FD patients with established ADA titers have been shown to have higher disease severity scores and exhibit accelerated renal functional decline compared to their ADA-negative counterparts [47,55] (Table 3).

## 3. Available Strategies to Mitigate and Overcome Immunogenicity

### 3.1. Can Dose Escalation Overcome ADAs Production in the Course of ERT?

Studies have shown that administering higher doses of ERT in ADA-positive patients may yield improved biochemical responses and potentially slow the progression of FD. This hypothesis is based on the principle that, if the administered ERT dose exceeds or saturates circulating ADA titers, a greater quantity of the enzyme can reach the lysosomes of target cells, thereby enhancing the therapeutic effect [56,57]. However, the available evidence indicates that dose escalation leads to inconsistent ADA responses and variable outcomes with respect to ADAs saturation. To investigate this further, Lenders et al. conducted a study involving 250 FD patients undergoing ERT. The researchers employed serum-mediated inhibition assays to detect ADAs and titration assays to quantify individual the inhibitory capacities of ADAs against agalsidase-α and agalsidase-β [58]. While switching from agalsidase-α to agalsidase-β led to ADA saturation in seven patients, sustained saturation was achieved in only two cases following dose escalation. Notably, comprehensive clinical and immunologic data were available only for a subset of participants, and the analyzed serum samples represented a heterogeneous FD male population, including individuals with late-onset disease and carriers of both missense and nonsense mutations, thereby limiting the generalizability of these findings [58]. Moreover, it can be hypothesized that the maximum clinically approved ERT dosage (1 mg/kg) may only be sufficient to achieve ADAs saturation in patients with low to moderate antibody titers, and since an increased dose of infused medication might lead to greater risk of adverse reactions along with higher overall cost, it should be carefully pondered in a real-life setting.

### 3.2. What Protocols Are Currently Being Assessed to Control Immunogenicity in FD?

To address immunogenicity arising during FD therapy, several strategies have been implemented:

- Immunoadsorption (IA) protocols based on non-specific IgG depletion have proven highly effective in various clinical settings, including myasthenia gravis, kidney and allogeneic hematopoietic stem cell transplantation, and autoimmune dilated cardiomyopathy [59,60,61]. While non-specific IA systems remove total Ig fractions, potentially weakening the overall humoral immune response, antigen-specific IA selectively depletes pathogenic antibodies without affecting other antibody populations or compromising the immune system [62]. In FD, the application of IA is still in the preclinical phase. Lenders et al. demonstrated in vitro that AGAL-specific ADAs can be selectively removed from the sera of FD patients. However, titers appear to recover rapidly, suggesting that high-frequency IA treatments would be necessary [63].
- The effectiveness of immunosuppressive (IS) therapy in improving outcomes of ERT in LSDs has been previously documented. It has been shown that immune tolerance can be achieved when IS treatment is initiated prior to or simultaneously with ERT. Clinical experience from other disorders treated with recombinant proteins demonstrates that, once ADAs develop, especially at high titers, they tend to persist, despite IS interventions [64]. Banugaria et al. reported that immune tolerance induction using rituximab, methotrexate, and intravenous immunoglobulin (IVIG) enhanced ERT efficacy in CRIM-negative infantile-onset Pompe disease [65]. However, it is noteworthy that, recently, anti-rituximab antibodies have been described in patients with membranous glomerulonephritis treated with rituximab and associated with less therapeutic effectiveness [66]. Dickson et al. documented benefits in a canine MPS-1 model using azathioprine and cyclosporine in combination with ERT [67]. However, because rituximab does not deplete memory B cells, additional administration of bortezomib effectively reduces ADA titers in infantile-onset Pompe disease [68,69]. Furthermore, pre-treatment with omalizumab has been shown to reduce IgE levels in FD patients [70]. While the global impact of antibodies on therapy remains partially understood, Garman et al. observed reduced antibody responses to agalsidase-β using 10 mg/kg methotrexate in a murine FD model [71]. More recent studies have shown that IS therapy, specifically with prednisolone, tacrolimus, and mycophenolate mofetil/mycophenolate acid, successfully eliminated antibody-mediated ERT inhibition in transplanted male FD patients. However, tapering IS therapy led to a recurrence of ERT inhibition, while higher IS doses correlated with lower ADA titers and reduced inhibition [72]. Therefore, IS therapy may serve as an effective approach to managing specific and clinically significant antibody responses over time. Nevertheless, the optimal IS regimen for preventing ADA formation in FD remains to be established, especially in patients with high levels of ERT inhibition, due to the potential adverse effects of IS agents. For these reasons, it might be advisable to modify the therapeutic scheme, i.e., prolonged half-life of infused enzymes, increased infusion frequencies with less enzyme concentrations, or reduced agalsidase-β infusion time, in order to minimize the need for IS interventions, even though the real effectiveness of these changes has not been definitively proven [72,73] (Table 4).

## 4. Conclusions

The development of immunogenicity during ERT in FD depends both on the molecular characteristics of the administered agent and on the intrinsic features of individual patients. Given the clinical and genetic heterogeneity of FD, large-scale observational and longitudinal real-world studies are required to elucidate the true impact of immunogenicity on disease progression, as well as to assess the efficacy of strategies implemented to manage it. Once ADAs develop, they tend to persist over time, with a fluctuating pattern that remains poorly understood. An expanding body of scientific evidence, both in FD and other LSDs, suggests that immunogenicity negatively affects therapeutic response. However, it remains difficult to establish a definitive correlation between ADA titer fluctuations and the clinical trajectory of the disease, or to determine clear implications for subsequent treatment strategies. The lack of an appropriate reference antibody makes it difficult to quantify the absolute concentration of ADAs in a single patient, which would be of extreme interest, as this information could be used to plan and optimize treatment. Therefore, it is essential to develop approaches aimed at limiting ADAs formation and promoting sustained immune tolerance to the therapeutic enzyme, particularly in treatment-naïve patients. Identifying individuals at high risk for ADA development before initiating ERT is a critical step toward achieving this goal. To facilitate robust data comparison while enhancing communication and information exchange among healthcare professionals, the implementation of validated assays for ADAs detection is strongly recommended to support clinical decision-making. This includes establishing reference laboratories with harmonized testing protocols and defining clinical biomarkers to assess the relationship between immunogenicity and disease evolution over time.

## Figures and Tables

**Table 1:** Currently approved ERTs for Fabry Disease.

	Agalsidase-α	Agalsidase-β	Pegunigalsidase-α
**Mechanism of action**	ERT from human embryonic cell line	ERT from CHO cells	ERT with pegylated enzyme from tobacco plant cells
**Mode and frequency of administration**	IV infusion, every 2 wks	IV infusion, every 2 wks	IV infusion, every 2 wks (4 wks in study)
**Treated population**	Wide, included null mutations	Wide, included null mutations	Wide, included null mutations
**IRRs**	IRRs are reported in 10% to 24% of pts *	70% of pts present IRRs *	Limited IRRs are reported *
**ADAs**	ADAs are reported as ranging from 20% to 55% [19,20,21] **	ADAs are reported as ranging from 73% to 91% [19,20,21] **	16% of pts develop ADAs

* As reported by the Italian Medicines Agency (Agenzia Italiana del Farmaco, AIFA); pts, patients; wks, weeks; enzyme replacement therapy (ERT); IV, intravenous; (IRRs)ADAs, anti-drug antibodies; infusion-related reactions (IRRs); CHO, Chinese hamster ovary. ** Reference [19], Wilcox et al., reports ADA titers from 820 patients; reference [20], Arakawa et al., reports IRRs from 129 patients.

**Table 2:** ADA formation according to the therapy used: key points.

**Production cell line and glycosylation patterns influence immunogenicity**	Agalsidase-α derives from human fibroblasts, whereas agalsidase-β is produced from CHO cells and contains non-human sialic acids that can trigger immune responses.
**Immunogenicity of the infused enzyme**	RCTs and PMS indicate a higher incidence of ADAs in patients treated with agalsidase-β vs. agalsidase-α.
**Cross-reactivity has implications for switching**	IgG ADAs against one ERT can cross-react with the other, with implications for switching therapies or interpreting immunogenicity data.
**Pegunigalsidase-α represents an option, but immunogenicity is not completely overcome**	Pegunigalsidase-α offers potential benefit in pts with existing ADAs against ERTs; however, it still induces ADAs and pre-existing anti-PEG or anti-AGAL ADAs can reduce its activity through immune complex formation.

pts, patients; ERT, enzyme replacement therapy; ADAs, anti-drug antibodies; CHO, Chinese hamster ovary; RCTs, randomized controlled trials; PMS, post-marketing surveillance.

**Table 3:** Impact of anti-agalsidase ADAs on FD biomarkers and ERT effectiveness: key points.

**ADA-Mediated Inhibition**	Neutralizing IgG1 and IgG4 ADAs block key epitopes on agalsidase, impairing M6P receptor uptake and reducing intracellular α-GAL activity.
**Reduced Biomarker Clearance**	High ADA titers correlate with persistent elevation of lysoGb3 and limited Gb3 clearance, especially in plasma and urine.
**Altered Pharmacodynamics**	ADAs form immune complexes that increase enzyme clearance via Fcγ receptor pathways, diminishing ERT efficacy.
**Clinical Impact**	ADA-positive patients, especially those with inhibitory ADAs, show worse clinical outcomes, accelerated renal decline, and increased disease severity.
**Non-Neutralizing ADA Effects**	Non-neutralizing ADAs may disrupt enzyme trafficking and conformation, affecting overall pharmacokinetics and therapeutic response.
**IARs**	ADA-positive patients are prone to develop IARs due to unclear pathogenic mechanisms.

Ig, immunoglobulin; ERT, enzyme replacement therapy; ADAs, anti-drug antibodies; MP6, mannose-6-phosphate; α-GAL, α-galactosidase A; LysoGb3, globotriaosylsphingosine; Gb3, globotriaosylceramide; immune adverse reactions (IAR).

**Table 4:** Strategies for immunogenicity control: key points.

**Dose escalation**	Dose escalation of ERT may help saturating ADA titers, but responses are variable, and this approach is likely effective in patients with low to moderate ADAs levels.
**Antigen-specific IA**	Antigen-specific IA can selectively deplete anti-AGAL antibodies in vitro. However, rapid ADA rebound would require frequent IA sessions, and its clinical application in FD remains investigational.
**IS therapy**	Early IS therapy may prevent ADA formation, as shown in Pompe and MPS-1. IS regimens include rituximab, methotrexate, IVIG, and bortezomib.
**Minimizing immunogenicity from the start**	Given the risks and long-term adverse effects of IS agents, it may be advisable to consider modifying the early therapeutic scheme to avoid the need for IS strategies later on.

ERT, enzyme replacement therapy; IS, immunosuppressive; ADAs, antidrug antibodies; FD, Fabry disease; IVIG, intravenous immunoglobulin.

## Data Availability

Not applicable.

## Abbreviations

ADAs	Antidrug antibodies
APCs	Antigen-presenting cells
CHO	Chinese hamster ovary
CRIM	Cross-reactive immunologic material
eGFR	Estimated glomerular filtration rate
ERT	Enzyme replacement therapy
FOS	Fabry Outcome Survey
Gb3	Globotriaosylceramide
GLA	α-galactosidase A
GSL	Glycosphingolipids
IA	Immunoadsorption
IAR	Immune adverse reaction
IRRs	Infusion-related reactions
IS	Immunosuppressive
IVIG	Intravenous immunoglobulin
LSD	Lysosomal storage disease
LysoGb3	Globotriaosylsphingosine
M6P	Mannose-6-phosphate
NGNA	N-glycolylneuraminic acid
OS	Oxidative stress
PAF	Platelet activating factor
PMS	Post-marketing surveillance
QoL	Quality of life
RCTs	Randomized controlled trials
α-AGAL	α-galactosidase A

## References

[1] Wanner C., Arad M., Baron R., Burlina A., Elliott P.M., Feldt-Rasmussen U., Fomin V.V., Germain D.P., Hughes D.A., Jovanovic A. (2018). European expert consensus statement on therapeutic goals in Fabry disease. Mol. Genet. Metab..

[2] Squillaro T., Antonucci I., Alessio N., Esposito A., Cipollaro M., Melone M.A.B., Peluso G., Stuppia L., Galderisi U. (2017). Impact of lysosomal storage disorders on biology of mesenchymal stem cells: Evidences from in vitro silencing of glucocerebrosidase (GBA) and alpha-galactosidase A (GLA) enzymes. J. Cell. Physiol..

[3] Lillo R., Graziani F., Franceschi F., Iannaccone G., Massetti M., Olivotto I., Crea F., Liuzzo G. (2023). Inflammation across the spectrum of hypertrophic cardiac phenotypes. Heart Fail. Rev..

[4] Lenders M., Brand E. (2021). Precision medicine in Fabry disease. Nephrol. Dial. Transplant..

[5] Azevedo O., Gago M.F., Miltenberger-Miltenyi G., Sousa N., Cunha D. (2020). Fabry disease therapy: State-of-the-art and current challenges. Int. J. Mol. Sci..

[6] Castelli V., Stamerra C.A., d’Angelo M., Cimini A., Ferri C. (2021). Current and experimental therapeutics for Fabry disease. Clin. Genet..

[7] Kant S., Atta M.G. (2020). Therapeutic advances in Fabry disease: The future awaits. Biomed. Pharmacother..

[8] Chiesi Global Rare Diseases, Protalix BioTherapeutics FDA Approval of ELFABRIO^®^ (pegunigalsidase alfa-iwxj) for the Treatment of Fabry Disease. *Press Release*, 2023. https://chiesirarediseases.com/media/fda-approval-of-elfabrio.

[9] Hughes D.A., Bichet D.G., Giugliani R., Hopkin R.J., Krusinska E., Nicholls K., Olivotto I., Feldt-Rasmussen U., Sakai N., Skuban N. (2023). Long-term multisystemic efficacy of migalastat on Fabry-associated clinical events. J. Med. Genet..

[10] van der Veen S.J., Vlietstra W.J., van Dussen L., van Kuilenburg A.B.P., Dijkgraaf M.G.W., Lenders M., Brand E., Wanner C., Hughes D., Elliott P.M. (2020). Predicting the development of antidrug antibodies against recombinant α-galactosidase A in male patients with classical Fabry disease. Int. J. Mol. Sci..

[11] Mauhin W., Lidove O., Masat E., Mingozzi F., Mariampillai K., Ziza J.M., Benveniste O. (2015). Innate and adaptive immune response in Fabry disease. JIMD Rep..

[12] Smid B.E., Hoogendijk S.L., Wijburg F.A., Hollak C.E., Linthorst G.E. (2013). A revised home treatment algorithm for Fabry disease: Influence of antibody formation. Mol. Genet. Metab..

[13] Arends M., Biegstraaten M., Wanner C., Sirrs S., Mehta A., Elliott P.M., Oder D., Watkinson O.T., Bichet D.G., Khan A. (2018). Agalsidase alfa versus agalsidase beta for the treatment of Fabry disease. J. Med. Genet..

[14] Limgala R.P., Jennelle T., Plassmeyer M., Boutin M., Lavoie P., Abaoui M., Auray-Blais C., Nedd K., Alpan O., Goker-Alpan O. (2019). Altered immune phenotypes in subjects with Fabry disease. Am. J. Transl. Res..

[15] Chu W., Chen M., Lv X., Lu S., Wang C., Yin L., Qian L., Shi J. (2025). Status and frontiers of Fabry disease. Orphanet J. Rare Dis..

[16] Rombach S.M., Smid B.E., Linthorst G.E., Dijkgraaf M.G., Hollak C.E. (2014). Natural course of Fabry disease and effectiveness of enzyme replacement therapy. J. Inherit. Metab. Dis..

[17] Garagiola I., Palla R., Peyvandi F. (2018). Risk factors for inhibitor development in severe hemophilia A. Thromb. Res..

[18] Bali D.S., Goldstein J.L., Banugaria S., Dai J., Mackey J., Rehder C., Kishnani P.S. (2012). Predicting CRIM status in Pompe disease. Am. J. Med. Genet. C.

[19] Wilcox W.R., Linthorst G.E., Germain D.P., Feldt-Rasmussen U., Waldek S., Richards S.M., Beitner-Johnson D., Cizmarik M., Cole J.A., Kingma W. (2012). Anti–α-galactosidase A antibody response to agalsidase beta: Fabry Registry data. Mol. Genet. Metab..

[20] Arakawa M., Ikeda Y., Otaka H., Iwashiro S. (2024). Long-term safety of enzyme replacement therapy with agalsidase alfa in patients with Fabry disease: Post-marketing extension surveillance in Japan. Mol. Genet. Metab. Rep..

[21] Gómez-Cerezo J.F., Fernández-Martín J., Barba-Romero M.Á., Sánchez-Martínez R., Hermida-Ameijeiras A., Camprodon-Gómez M., Ortolano S., Lopez-Rodriguez M.A. (2025). Immunogenicity of ERT in Fabry disease. Orphanet J. Rare Dis..

[22] Keslová-Veselíková J., Hůlková H., Dobrovolný R., Asfaw B., Poupetová H., Berná L., Sikora J., Goláň L., Ledvinová J., Elleder M. (2008). Replacement of α-galactosidase A in Fabry disease: Effect on fibroblasts and patient tissues. Virchows Arch..

[23] Deegan P.B. (2012). Fabry disease, enzyme replacement therapy and the significance of antibody responses. J. Inherit. Metab. Dis..

[24] Warnock D.G., Wallace E.L. (2024). Response to commentary: Head-to-head trial of pegunigalsidase alfa versus agalsidase beta in patients with Fabry disease and deteriorating renal function: Results from the 2-year randomised phase III BALANCE study—Determination of immunogenicity. J. Med. Genet..

[25] Linthorst G.E., Hollak C.E., Donker-Koopman W.E., Strijland A., Aerts J.M. (2004). Enzyme therapy for Fabry disease: Neutralizing antibodies toward agalsidase alpha and beta. Kidney Int..

[26] Bork K., Horstkorte R., Weidemann W. (2009). Increasing the sialylation of therapeutic glycoproteins: The potential of the sialic acid biosynthetic pathway. J. Pharm. Sci..

[27] Ghaderi D., Taylor R.E., Padler-Karavani V., Diaz S., Varki A. (2010). Implications of the presence of N-glycolylneuraminic acid in recombinant therapeutic glycoproteins. Nat. Biotechnol..

[28] Sakuraba H., Murata-Ohsawa M., Kawashima I., Tajima Y., Kotani M., Ohshima T., Chiba Y., Takashiba M., Jigami Y., Fukushige T. (2006). Comparison of the effects of agalsidase alfa and agalsidase beta on cultured human Fabry fibroblasts and Fabry mice. J. Hum. Genet..

[29] Bekri S., Mehta A., Beck M., Sunder-Plassmann G. (2006). Importance of glycosylation in enzyme replacement therapy. Fabry Disease: Perspectives from 5 Years of FOS.

[30] Meghdari M., Gao N., Abdullahi A., Stokes E., Calhoun D.H. (2015). Carboxyl-terminal truncations alter α-galactosidase A activity. PLoS ONE.

[31] Vedder A.C., Breunig F., Donker-Koopman W.E., Mills K., Young E., Winchester B., Berge I.J.T., Groener J.E., Aerts J.M., Wanner C. (2008). Treatment of Fabry disease with different dosing regimens of agalsidase. Mol. Genet. Metab..

[32] Vedder A.C., Linthorst G.E., Houge G., Groener J.E., Ormel E.E., Bouma B.J., Aerts J.M., Hirth A., Hollak C.E. (2007). Treatment of Fabry disease: Comparative trial of agalsidase alfa or beta at 0.2 mg/kg. PLoS ONE.

[33] Keating G.M. (2012). Agalsidase alfa: A review of its use in Fabry disease. BioDrugs.

[34] Lenders M., Menke E.R., Rudnicki M., Cybulla M., Brand E. (2025). Neutralizing antibodies and pharmacokinetics of pegunigalsidase alfa. BioDrugs.

[35] Lee C.S., Kulkarni Y., Pierre V., Maski M., Wanner C. (2024). Adverse impacts of PEGylated protein therapeutics. BioDrugs.

[36] Lenders M., Pollmann S., Terlinden M., Brand E. (2022). Pre-existing antibodies in Fabry disease: Reduced affinity for pegunigalsidase alfa. Mol. Ther. Methods Clin. Dev..

[37] Shire Human Genetic (2001). Replagal (Agalsidase Alfa).

[38] Barbey F., Livio F. (2006). Safety of enzyme replacement therapy. Fabry Disease: Perspectives from 5 Years of FOS.

[39] Genzyme Corporation (2003). Fabrazyme (Agalsidase Beta).

[40] Berry L., Walter J., Johnson J., Alton J., Powers J., Llòria X., Koulinska I., McGee M., Laney D. (2024). Patient-reported experience with Fabry disease. Orphanet J. Rare Dis..

[41] Finkelman F.D. (2007). Anaphylaxis: Lessons from mouse models. J. Allergy Clin. Immunol..

[42] Matucci A., Vultaggio A., Nencini F., Maggi E. (2020). Anaphylactic reactions to biological drugs. Curr. Opin. Allergy Clin. Immunol..

[43] Lenders M., Brand E. (2018). Effects of ERT and antidrug antibodies in Fabry disease. J. Am. Soc. Nephrol..

[44] Biesenbach P., Kain R., Derfler K., Perkmann T., Soleiman A., Benharkou A., Druml W., Rees A., Säemann M.D. (2014). Long-Term Outcome of Anti-Glomerular Basement Membrane Antibody Disease Treated with Immunoadsorption. PLoS ONE.

[45] Ohashi T., Iizuka S., Ida H., Eto Y. (2008). Reduced α-Gal A enzyme activity in Fabry fibroblast cells and Fabry mice tissues induced by serum from antibody positive patients with Fabry disease. Mol. Genet. Metab..

[46] Bénichou B., Goyal S., Sung C., Norfleet A.M., O’Brien F. (2009). A retrospective analysis of the potential impact of IgG antibodies to agalsidase β on efficacy during enzyme replacement therapy for Fabry disease. Mol. Genet. Metab..

[47] van der Veen S.J., van Kuilenburg A.B.P., Hollak C.E.M., Kaijen P.H.P., Voorberg J., Langeveld M. (2019). Antibodies against recombinant alpha-galactosidase A in Fabry disease: Subclass analysis and impact on response to treatment. Mol. Genet. Metab..

[48] Nowak A., Mechtler T.P., Desnick R.J., Kasper D.C. (2017). Plasma LysoGb3 as a biomarker. Mol. Genet. Metab..

[49] Scharnetzki D., Stappers F., Lenders M., Brand E. (2020). Epitope mapping of neutralizing antibodies in Fabry disease. Mol. Genet. Metab..

[50] Ronco P., Debiec H. (2015). Advances in membranous nephropathy. Lancet.

[51] Rombach S.M., Aerts J.M., Poorthuis B.J., Groener J.E., Donker-Koopman W., Hendriks E., Mirzaian M., Kuiper S., A Wijburg F., Hollak C.E.M. (2012). Long-term effect of antibodies on lysoGb3 reduction. PLoS ONE.

[52] Lenders M., Canaan-Kühl S., Krämer J., Duning T., Reiermann S., Sommer C., Stypmann J., Blaschke D., Üçeyler N., Hense H.-W. (2016). Patients with Fabry Disease after Enzyme Replacement Therapy Dose Reduction and Switch–2-Year Follow-Up. J. Am. Soc. Nephrol..

[53] Stappers F., Scharnetzki D., Schmitz B., Manikowski D., Brand S.M., Grobe K., Lenders M., Brand E. (2020). Neutralising anti-drug antibodies in Fabry disease can inhibit endothelial enzyme uptake and activity. J. Inherit. Metab. Dis..

[54] Ramaswami U., Bichet D.G., Clarke L.A., Dostalova G., Fainboim A., Fellgiebel A., Forcelini C.M., Haack K.A., Hopkin R.J., Mauer M. (2019). Low-dose agalsidase beta in pediatric males. Mol. Genet. Metab..

[55] Lenders M., Stypmann J., Duning T., Schmitz B., Brand S.M., Brand E. (2016). Serum-Mediated Inhibition of Enzyme Replacement Therapy in Fabry Disease. J. Am. Soc. Nephrol..

[56] Lenders M., Schmitz B., Brand S.M., Foell D., Brand E. (2018). Drug-neutralizing antibodies during infusion. J. Allergy Clin. Immunol..

[57] Lenders M., Neußer L.P., Rudnicki M., Nordbeck P., Canaan-Kühl S., Nowak A., Cybulla M., Schmitz B., Lukas J., Wanner C. (2018). Dose-Dependent Effect of Enzyme Replacement Therapy on Neutralizing Antidrug Antibody Titers and Clinical Outcome in Patients with Fabry Disease. J. Am. Soc. Nephrol..

[58] Lenders M., Brand E. (2022). Dose escalation and antidrug antibodies. Front. Immunol..

[59] Lazaridis K., Baltatzidou V., Tektonidis N., Tzartos S.J. (2020). Antigen-specific immunoadsorption of MuSK autoantibodies as a treatment of MuSK-induced experimental autoimmune myasthenia gravis. J. Neuroimmunol..

[60] Handisurya A., Worel N., Rabitsch W., Bojic M., Pajenda S., Reindl-Schwaighofer R., Winnicki W., Vychytil A., Knaus H.A., Oberbauer R. (2020). Antigen-Specific Immunoadsorption With the Glycosorb^®^ ABO Immunoadsorption System as a Novel Treatment Modality in Pure Red Cell Aplasia Following Major and Bidirectional ABO-Incompatible Allogeneic Hematopoietic Stem Cell. Front. Med..

[61] Pavenski K., Bucholz M., Cheatley P.L., Krok E., Anderson M., Prasad G.R., Qureshi M.A., Meliton G., Zaltzman J. (2020). The First North American Experience Using Glycosorb Immunoadsorption Columns for Blood Group-Incompatible Kidney Transplantation. Can. J. Kidney Health Dis..

[62] Hamilton P., Harris R., Mitra S., Karkar A. (2019). Chapter Four—Immunoadsorption techniques and its current role in the intensive care unit, aspects in continuous renal replacement therapy. Aspects in Continuous Renal Replacement Therapy.

[63] Lenders M., Scharnetzki D., Heidari A., Di Iorio D., Wegner S.V., Brand E. (2021). Generation and Characterization of a Polyclonal Human Reference Antibody to Measure Anti-Drug Antibody Titers in Patients with Fabry Disease. Int. J. Mol. Sci..

[64] Poelman E., Hoogeveen-Westerveld M., van den Hout J.M.P., Bredius R.G.M., Lankester A.C., Driessen G.J.A., Kamphuis S.S.M., Pijnappel W.W.M., van der Ploeg A.T. (2019). Immunomodulation in infantile Pompe disease with high antibody titers. Orphanet J. Rare Dis..

[65] Banugaria S.G., Prater S.N., Patel T.T., Dearmey S.M., Milleson C., Sheets K.B., Bali D.S., Rehder C.W., Raiman J.A.J., A Wang R. (2013). Algorithm for the early diagnosis and treatment of patients with cross reactive immunologic material-negative classic infantile pompe disease: A step towards improving the efficacy of ERT. PLoS ONE.

[66] Allinovi M., Teisseyre M., Accinno M., Finocchi C., Esnault V.L.M., Cremoni M., Mazzierli T., Lazzarini D., Casiraghi M.A., Fernandez C. (2025). Anti-rituximab antibodies in membranous nephropathy. Kidney Int. Rep..

[67] Dickson P., Peinovich M., McEntee M., Lester T., Le S., Krieger A., Manuel H., Jabagat C., Passage M., Kakkis E.D. (2008). Immune tolerance improves the efficacy of enzyme replacement therapy in canine mucopolysaccharidosis I. J. Clin. Investig..

[68] Choi S.J., Yi J.S., Lim J.A., Tedder T.F., Koeberl D.D., Jeck W., Desai A.K., Rosenberg A., Sun B., Kishnani P.S. (2023). Successful AAV8 readministration: Suppression of capsid-specific neutralizing antibodies by a combination treatment of bortezomib and CD20 mAb in a mouse model of Pompe disease. J. Gene Med..

[69] Chen H.A., Hsu R.H., Fang C.Y., Desai A.K., Lee N.C., Hwu W.L., Tsai F.-J., Kishnani P.S., Chien Y.-H. (2024). Optimizing treatment outcomes: Immune tolerance induction in Pompe disease patients undergoing enzyme replacement therapy. Front. Immunol..

[70] DuBuske I., Schmidlin K., Bernstein J.A. (2021). Successful desensitization to agalsidase beta anaphylaxis with omalizumab. Ann. Allergy Asthma Immunol..

[71] Garman R.D., Munroe K., Richards S.M. (2004). Methotrexate reduces antibody responses to recombinant human alpha-galactosidase A therapy in a mouse model of Fabry disease. Clin. Exp. Immunol..

[72] Lenders M., Oder D., Nowak A., Canaan-Kühl S., Arash-Kaps L., Drechsler C., Schmitz B., Nordbeck P., Hennermann J.B., Kampmann C. (2017). Impact of immunosuppressive therapy on therapy-neutralizing antibodies in transplanted patients with Fabry disease. J. Intern. Med..

[73] Mignani R., Americo C., Aucella F., Battaglia Y., Cianci V., Sapuppo A., Lanzillo C., Pennacchiotti F., Tartaglia L., Marchi G. (2024). Reduced infusion time of agalsidase beta: Low antibody formation. Orphanet J. Rare Dis..

